# PATTERNS OF SEDENTARY BEHAVIORS IN OLDER ADULTS IN CHINESE CARE HOMES: A LATENT PROFILE ANALYSIS

**DOI:** 10.1093/geroni/igae098.0243

**Published:** 2024-12-31

**Authors:** Qi Li, Sihan Dong, Xueying Liu, Mengjiao Xu, Yuting Song

**Affiliations:** Qingdao University, Qingdao, Shandong, China (People’s Republic); Qingdao University, Qingdao, Shandong, China (People’s Republic); Qingdao University, Qingdao, Shandong, China (People’s Republic); Qingdao University, Qingdao, Shandong, China (People’s Republic); Qingdao University, Qingdao, Shandong, China (People’s Republic)

## Abstract

Sedentary behaviors remain common among older adults. Previous studies in this area are mostly focused on community settings and used variable-centered approach. In this study, we aimed to identify the pattern of sedentary behaviors among older adults in residential care homes in China. This cross-sectional analysis used data from 283 older adults who lived in 11 residential care homes in China. Eligible participant aged 65 years or older, had lived in the current care home for ≥3 months, and was able to communicate verbally or in writing. Sedentary behaviors were assessed with the Elderly Sedentary Behavior Questionnaire. Participants self-reported the average daily time spent in the past week on each of eight behaviors, such as watching TV, socializing, and doing nothing. Patterns of sedentary behaviors were identified using latent profile analysis and verified by associating with depressive symptoms using a regression model. The average daily sedentary time of older people was 11.6 hours (SD=2.6). Four patterns of sedentary behaviors emerged, which we labeled the high sedentism group (23%), the balanced group (50%), the high mentally-active group (8%), and the high mentally-passive group (19%). Compared to older adults in the balanced group, those in the high sedentism group and high mentally-passive group had higher risks of depressive symptoms and those in the high mentally-active group had lower risks of depressive symptoms. Targeted interventions are warranted for older adults of different patterns of sedentary behaviors, particularly older adults in the high sedentism group and high mentally-passive group.

